# TNF-α promotes gallbladder cancer cell growth and invasion through autocrine mechanisms

**DOI:** 10.3892/ijmm.2014.1711

**Published:** 2014-03-24

**Authors:** GUANGWEI ZHU, QIANG DU, XIAOQIAN WANG, NANHONG TANG, FEIFEI SHE, YANLING CHEN

**Affiliations:** 1 Department of Hepatobiliary Surgery, The Affiliated Union Hospital of Fujian Medical University, Fuzhou, Fujian, P.R. China; 2 Key Laboratory of Ministry of Education for Gastrointestinal Cancer, Fuzhou, Fujian, P.R. China; 3 Key Laboratory of Tumor Microbiology, School of Basic Medical Sciences, Fujian Medical University, Fuzhou, Fujian, P.R. China

**Keywords:** gallbladder cancer cell, tumor necrosis factor-α, siRNA, autocrine, proliferation, invasion, apoptosis

## Abstract

Tumor necrosis factor-α (TNF-α) has been suggested to be a putative tumor promoter gene, and autocrine of TNF-α expression has been found in colon cancer and ovarian cancer. As the role of autocrine TNF-α in human gallbladder cancer has not yet been elucidated, the present study examined the expression of TNF-α in gallbladder cancer-derived cell lines. Based on the data, TNF-α mRNA and TNF-α protein expression differed significantly different between the cell lines. In addition, using siRNA targeting TNF-α, the vector, pGPU-GFP-siTNF-α, was constructed and then transfected into the SGC-996 cells (gallbladder cancer cell line) which express high levels of endogenous TNF-α. *In vitro* experiments indicated that the silencing of TNF-α in the SGC-996 cells significantly suppressed proliferation and invasion. However, apoptosis was not induced by the silencing of TNF-α. Furthermore, we traced the mechanisms underlying these effects and found that the silencing of TNF-α affected the TNF-α-AKT-NF-κB-Bcl-2 pathway in the SGC-996 cells. Our data provide evidence that autocrine TNF-α plays a role as a tumor promoter gene in gallbladder cancer cells, possibly by promoting proliferation and invasion through autocrine mechanisms.

## Introduction

Gallbladder cancer is a relatively rare but highly lethal disease and is the most common cancer of the biliary tract and the seventh most common gastrointestinal carcinoma (1). Gallbladder cancer is a highly invasive and aggressive disease with a dismal prognosis and the 5-year survival rate for all stages of gallbladder cancer is approximately 5% (2,3). Gallbladder cancer has a very poor prognosis due to its invasive and aggressive characteristics which are determined by various factors. One of these factors is the tumor microenviroment. Numerous studies have confirmed that tumor necrosis factor-α (TNF-α) is a key cytokine amongst all cytokines of the tumor microenvironment which promote tumor cell proliferation and invasion. In a previous study, in an orthotopic mouse model of pancreatic cancer, treatment with anti-TNF-α antibody resulted the reduction of tumor growth and metastasis (4). In studies on animal thoracic neoplasms, dermatoma and gastrointestinal cancer, the association between tumor cell growth and metastasis and the amount of TNF-α in the tumor microenvironment has been demonstrated (5–7). In patients with malignant tumors of the prostate, it has also been shown that there is a correlation the amount of TNF-α and the degree of malignancy, recurrence, metastasis and prognosis (8). Studies have investigated the effects of TNF-α on tumor cell metastasis, demonstrating that TNF-α enhances the invasive capacity of cancer cells (9,10).

However, the specific mechanisms responsible for TNF-α promoting the progression of malignant tumors have not been elucidated. Certain studies have found that TNF-α promotes a variety of inflammatory cytokines and chemokines, thus affecting the formation and development of tumor blood vessels, promoting tumor invasion and metastasis (11). TNF-α activates associated cell signaling pathways through the activation of transcription factors and related genes, consequently affecting the activity of tumor cells, promoting tumor cell proliferation (12). TNF-α directly leads to gene damage, mutation, amplification of DNA, consequently affecting tumor development (13–15). In addition, TNF changes the function of immune cells, promoting tumor progression (16).

However, there are few reports on the function of tumor-derived TNF-α. Studies on colon and ovarian cancer have found that tumor-derived TNF-α plays an important role in tumor progression (5,11).

However, the specific mechanisms responsible for TNF-α promoting cancer cell growth and invasion are largely unknown. Studies have demonstrated that TNF-α is a key cytokine amongst all cytokines of the tumor microenvironment. Hagemann *et al* (17) demonstrated that in epithelial tumors, TNF-α stimulates matrix metalloproteinase (MMP) secretion, thereby promoting tumor cell invasion. Kulbe *et al* (18) found that in ovarian cancer cells, TNF-α stimulates IL-8, monocyte chemotactic protein-1 (MCP-1) and chemokine receptor expression, thus enhancing tumor cell invasion and metastasis. Chua *et al* (19) demonstrated that TNF-α enhances epithelial-mesenchymal transition in mammary epithelial cells. Another study also found that TNF-α induces the expression of vascular endothelial growth factor (VEGF), thus promoting microvascularization (20).

Tumor cell-derived TNF-α is a important factor produced by tumor cells and plays a key role in the tumor microenviroment (21). Moreover, TNF-α may even promote tumor growth at lower levels (22). Colon cancer cell-derived TNF-α plays an important role in promoting proliferation through autocrine mechanisms found in the tumor microenvironment (5). In ovarian cancer, it has been shown that tumor-derived TNF-α plays an important role in promoting invasion and metastasis (11,23,24).

However, whether gallbladder cancer cells produce autocrine TNF-α, and whether gallbladder cancer cell-derived TNF-α affects the biological behavior of the cells, remain unresolved issues. Thus, in the present study, we examined various gallbladder cancer cell lines expressing different levels of TNF-α in order to determine the effects of TNF-α on gallbladder cancer proliferation, invasion, metastasis and apoptosis, as well as the underlying mechanisms involved.

## Materials and methods

### Cell culture

The gallbladder cancer cell line, SGC-996, was provided by the Tumor Cytology Research Unit, Medical College, Tongji University, Shanghai, China. NOZ cells were obtained from the Health Science Research Resources Bank in Japan, and they were isolated from ascites derived from a 48-year-old female patient with gallbladder cancer (25). Both the cell lines were cutured in Dulbecco’s modified Eagle’s medium (DMEM) supplemented with 10% fetal bovine serum (FBS). All the cells were incubated at 37°C under 95% air and 5% CO_2_.

### Reverse transcription-polymerase chain reaction (RT-PCR)

Total RNA was extracted from the gallbladder cells grown in 6-well plates using TRIzol reagent (Invitrogen, Carlsbad, CA, USA) according to the manufacturer’s instructions. cDNA was synthesized using the AVM First Strand cDNA synthesis kit (Invitrogen). The primers for TNF-α and β-actin were synthesized according to primer design principles. TNF-α yielded a 443 bp product, and the sequences of the primers were as follows: forward, 5′-AGTGACAAGCCTGTAGCCC-3′ and reverse, 5′-GCAATGATCCCAAAGTAGACC-3′; TFN receptor 1 (TNFR1) yielded a 223 bp product, and the sequences of the primers were as follows: forward, 5′-TGCCA GGAGAAACAGAACA-3′ and reverse, 5′-AACCAA TGAAGAGGAGGGAT-3′. β-actin yielded a 254 bp product, and the sequences of the primers were as follows: forward, 5′-CTGTCTGGCGGCACCACCAT-3′ and reverse, 5′-GCAA CTAAGTCATAGTCCGC-3′. RT-PCR was performed under the following conditions: 30 cycles of denaturation at 94°C for 30 sec, annealing at 55°C for 30 sec, and extension at 72°C for 1 min fllowed by 10 min for final extension at 72°C. The data of TNF-α were normalized relative to the expression of β-actin mRNA expression in the respective samples.

### Western blot analysis

The cells were washed twice with cold phosphate-buffered saline (PBS) and then incubated on ice with 250 μl of RIPA buffer with 2.5 μl phenylmethylsulfonyl fluoride (PMSF) for 20 min. The cells were collected and centrifuged at 13,000 rpm for 10 min at 4°C. The protein concentrations of the cell lysates were measured in duplicate using a BCA Protein assay kit (Beyotime Institute of Biotechnology, Shanghai, China). The proportion of protein lysates and 6X loading buffer according to the ratio of 4:1 were mixed and then boiled for 5 min at 100°C. Equal amounts of total protein were resolved by sodium dodecyl sulfate (SDS 10%)-polyacrylamide gel electrophoresis and transferred onto polyvinylidene fluoride (PVDF) membranes. The PVDF membranes were then blocked with 5% non-fat milk in Tris-buffered saline with Tween-20 (TBST) for 2 h. The diluted primary antibodies, including polyclonal goat anti-human TNFR1 antibody (1:1,000), monoclonal mouse anti-human TNF-α (1:500) (both from Santa Cruz Biotechnology, Inc. Santa Cruz, CA, USA), monoclonal mouse anti-human AKT (1:500), monoclonal mouse anti-human p-AKT (1:500), monoclonal mouse anti-human nuclear factor-κB (NF-κB) (p65) (1:500), monoclonal mouse anti-human p-NF-κB (p-p65) (1:500) (all from Cell Signaling Technology, Danvers, MA, USA), monoclonal mouse anti-human Bcl-2 (1:500), monoclonal mouse anti-human Bax (1:500) and β-actin (1:1,500) (all from Santa Cruz Biotechnology, Inc.) were then incubated with the membranes overnight at 4°C. The appropriate secondary antibody conjugated with horseradish peroxidase diluted in TBST was added for 2 h at room temperature. Using a chemiluminescence western blot immunodetection kit (Invitrogen), we tested immunoreactivity according to the manufacturer’s instructions and recorded the data on hyperfine-ECL detection film. The amounts of TNF-α and TNFR1 protein were semiquantified as ratios to β-actin suggested on each gel.

### TNF-α siRNA plasmid construction and transfection

Suitable siRNA target sequences were found in the human TNF-α sequence. According to the design guidelines of siRNA and the literature (5), DNA template oligonucleotides corresponding to siRNA sequences were synthesized as follows: 5′-GCGTGGAGCTGAGAGATAA-3′. A small hairpin RNA (shRNA) of human TNF-α in a pGPU-GFP-neo gene transfer vector encoding a green fluorescent protein (GFP) sequence was constructed by GenePharma Co., Ltd. (Shanghai, China). The plasmids were verified by DNA sequencing. Corresponding sequences (C-N) for the negative contols (NC) were also provided by GenePharma Co., Ltd. The SGC-996 cells were cultured in DMEM medium supplemented with 10% FBS. When the cells were at approximately 90% confluency, we transfected the plasmids into the cells. The cells were transfected using Lipofectamine 2000 (Invitrogen) according to the manufacturer’s instructions. The transfection efficiency was quantified by determining the percentage of cells that were GFP-positive using a microscope (Fig. 3A and B). The culture medium was replaced with a selection medium containing G418 at concentrations of 400 μg/ml (Alexis Biochemicals, San Diego, CA, USA) 72 h later. When we obtained the stably transfected cells, the cells were continuously maintained in 200 μg/ml of G418 (Fig. 3C and D).

### Ezyme-linked immunosorbent assay (ELISA)

To analyze autocrine TNF-α in the untransfected SGC-996 cells, TNF-α small interfering (siRNA)-transfected SGC-996 cells and the SGC-996 negative control (NC)-transfected cells, these cells were seeded into plates at a density of 3×10^6^ cells/well with 4 ml of DMEM medium supplemented with 10% FBS. The amount of autocrine TNF-α in the cells was determined after 1, 2, 3, 4 and 5 days by ELISA. ELISA was performed to detect TNF-α in the cell culture supernatants of the untransfected SGC-996 cells, the TNF-α siRNA-transfected SGC-996 cells and the SGC-996NC-transfected cells using a TNF-α (H) ELISA kit (Wuhan Boster Biological Technology, Ltd., Wuhan, China). Two hundred microliters of supernatant were added to each well. ELISA was perforemd according to the manufacturer’s instructions. The sensitivity of the assays was 7.8 pg/ml. The absorbance was detected at 450 nm. Each plate test was repeated 3 times.

### Cell proliferation assay

To analyze cell proliferation, the untransfected SGC-996, TNF-α siRNA-transfected SGC-996 and SGC-996NC-transfected cells were seeded into 96-well plates at a density of 10^3^ cells/well with 100 μl of DMEM medium supplemented with 10% FBS. The proliferative activity was determined after 1, 2, 3, 4 and 5 days by the addition of 10 μl of sterile 3-(4,5-dimethylthiazol-2-yl)-2,5-diphenyltetrazolium bromide (MTT) (5 mg/ml; Sigma, St. Louis, MO, USA) to each well. The reaction was terminated after 4 h of incubation at 37°C by the addition of 100 μl of dimethyl sulfoxide (DMSO; Sigma). The optical density (OD) value was obtained by measuring absorbance at a wavelength of 570 nm. Each well test was repeated 6 times.

### In vitro cell migration assay

Cell motility was assayed using Transwells (24-well format) with 8 μm pore polycarbonate membranes (BD Biosciences, San Jose, CA, USA). The lower side of the membranes was covered with 5 μg fibronectin (BD Biosciences). To test cell motility induced by TNF-α siRNA, the treated or untreated SGC-996 cells (2×10^5^) in 200 μl of DMEM medium with 2.5% FBS were placed in the upper chamber. The lower chamber was filled with 700 μl DMEM medium with 10% FBS as the chemoattractant. The migration chamber was incubated for 8 h at 37°C and 5% CO_2_. The cells on the upper surface of the membrane were removed by gentle scrubbing with a cotton swab. Membranes were fixed in a stationary liquid of 95% ethanol and 5% acetic acid for 30 min and stained with hematoxylin and eosin (H&E). The number of cells on the lower surface of the membrane in 5 random visual fields (×400) was then counted using a bright field light microscope. Each assay was repeated in triplicate.

### In vitro cell invasion assay

For invasion assays, Transwells (24-well format) with 8 μm polycarbonate membranes (BD Biosciences) were used. Briefly, the upper side of the membranes was coated with Matrigel matrix (20 μg/well) and the membranes were then air-dried for 1 h of incubation 37°C. The lower side of the membranes was coated with 5 μg fibronectin (BD Biosciences). Other experimental procedures were the same as those for the migration assay.

### Flow cytometric analysis

To determine the apoptosis induced by TNF-α siRNA, the treated or untreated SGC-996 cells were seeded (5×10^5^/well) in 6-well plates in DMEM medium with 10% FBS for 48 h to collect the cells and stained using the Annexin V-PE/7-aminoactinomycin D kit (KeyGen Biotech, Nanjing, China) according the manufacturer’s instructions, and analyzed using a Becton-Dickinson FACSCalibur.

### Ststistical analysis

Data were analyzed using GraphPad Prism 5 software. Analysis of variance was conducted followed by one-way ANOVA or an unpaired t-test. The data are expressed as the means ± standard deviation (SD). A P-value <0.05 was considered to indicate a statistically significant difference.

## Results

### mRNA expression of TNF-α and TNFR1 in the NOZ and SGC-996 cells

**Figure 1:**
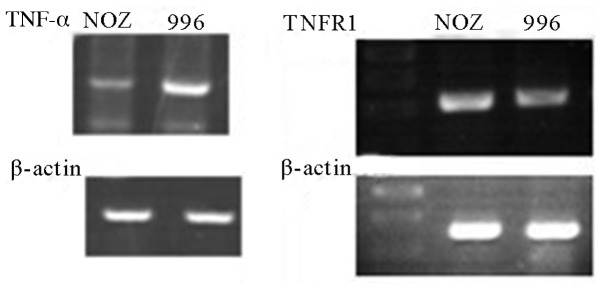
mRNA expression of tumor necrosis factor-α (TNF-α) and TNF receptor 1 (TNFR1) in the NOZ and SGC-996 (996) cells. The TNF-α mRNA expression level in the NOZ cells was lower than that in the SGC-996 cells.

We analyzed the mRNA expression of TNF-α and TNFR1 in the NOZ and SGC-996 cells. Using RT-PCR, we detected the mRNA expression of TNF-α and TNFR1 in both cell lines (Fig. 1). The TNF-α mRNA expression level in the NOZ cells was lower than that in the SGC-996 cells (Fig. 1). However, the mRNA levels of TNFR1 were similar between the NOZ and SGC-996 cells (Fig. 1). Thus, we used the SGC-996 cells to further examine the role of autocrine TNF-α.

### Protein expression of TNFR1 and TNF-α in the NOZ and SGC-996 cells

**Figure 2:**
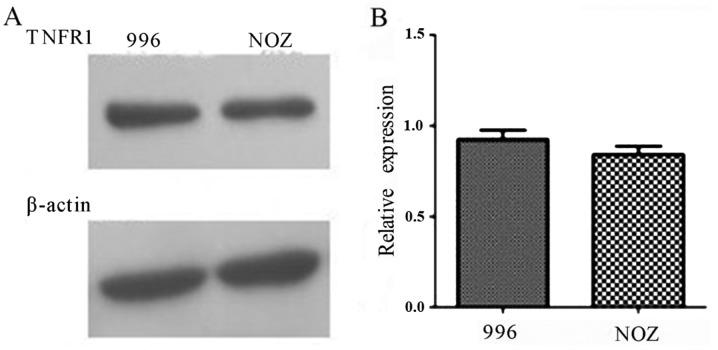

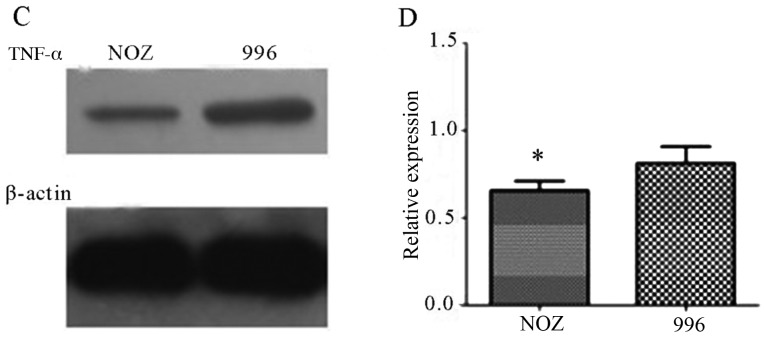
Protein expression of (A) tumor necrosis factor (TNF) receptor 1 (TNFR1) and (C) TNF-α in the SGC-996 (996) and NOZ cells detected by western blot analysis. TNFR1 and TNF-α protein expression was semiquantified by western blot analysis. The β-actin gene was used as an internal control. (B and D) The densitometric value for both groups were normalized to the internal control and relative expression with the following equation: normalization of both groups/the first normalized value. Data represent the means ± standard deviation (SD), n=3; ^*^P<0.05.

**Figure 3:**
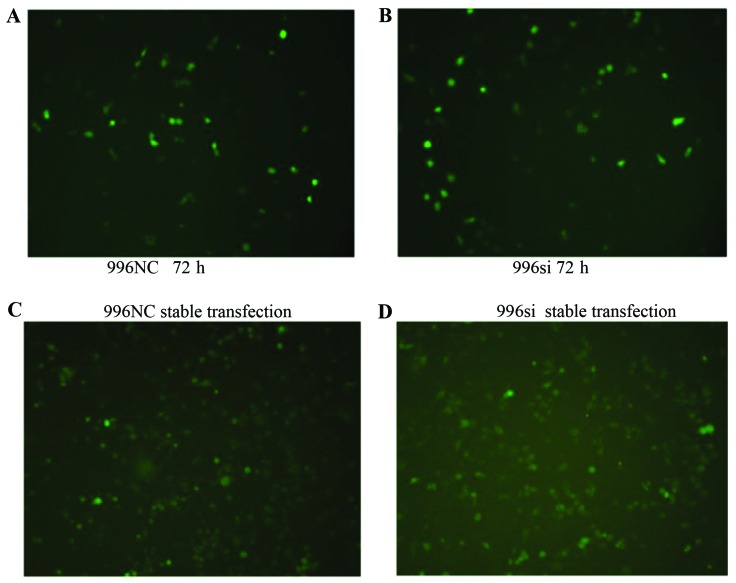
Tumor necrosis factor-α (TNF-α) siRNA plasmid construction and transfection. (A and B) Expression of mock vector green fluorescent protein (GFP) and siTNF-α-GFP was observed at 72 h after transfection by fluorescence microscopy. (C and D) Stable expression of mock vector GFP and siTNF-α-GFP was observed after a screening test by G418 using a fluorescence microscope. (A–D) Fluorescent light, ×200. 996si, SGC-996 cells transfected with siRNA targeting TNF-α; 996NC, SGC-996 cells transfected with negative control.

We then determined the TNFR1 and TNF-α protein expression in the NOZ and SGC-996 cells by western blot analysis. As expected, the protein expression of TNFR1 and TNF-α was detected in both cell lines. We observed no difference in the TNFR1 protein expression levels in the 2 cell lines by western blot analysis (Fig. 2); however, the TNF-α protein expression level in the NOZ cells was lower than that in the SGC-996 cells.

### mRNA and protein expression of TNF-α after obtaining stably transfected SGC-996 (SGC-996si) cells

**Figure 4:**
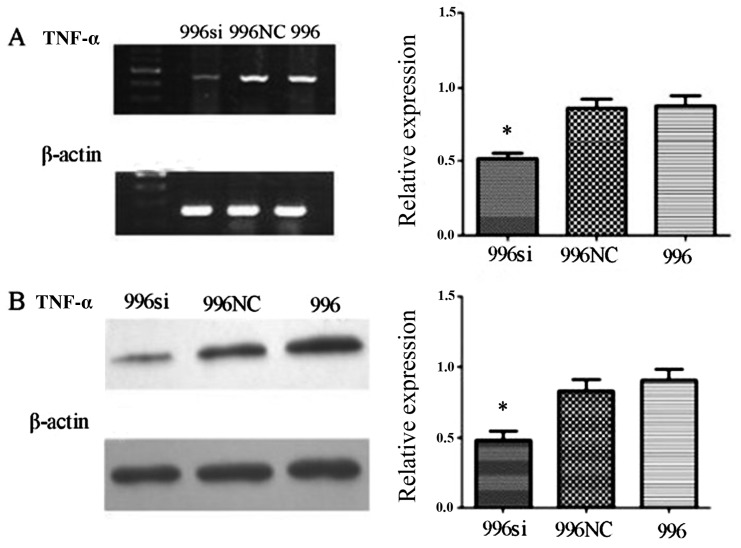
(A) RT-PCR and (B) western blot analysis indicated that the mRNA and protein levels of autocrine tumor necrosis factor-α (TNF-α) were significantly inhibited in the SGC-996si cells. Semiquantitative analysis showed that the TNF-α mRNA and protein levels in the SGC-996si group were markeldy suppressed. The densitometric value for both groups was normalized to the internal control and relative expression with the following equation: normalization of both groups/the first normalized value. Data represent the means ± standard deviation (SD), n=3; ^*^P<0.05. 996si, SGC-996 cells transfected with siRNA targeting TNF-α; 996NC, SGC-996 cells transfected with negative control; 996, untransfected SGC-996 cells.

We used an RNAi-mediated method to silence TNF-α in order to characterize the biological effects of TNF-α in the SGC-996 cells. The DNA sequencing results verified that TNF-α siRNA plasmid construction was successful. We obtained stably transfected cells using G418 after 2 weeks, as shown by a screening test. RT-PCR revealed a decrease in TNF-α mRNA levels in the siRNA-transfected cells, while the levels of the β-actin gene maintained relatively unaltered and mock transfection or transfection with the C-N/siRNA vector had no effect on TNF-α mRNA expression (Fig. 4A). We obtained stably transfected SGC-996 cells by siRNA targeting TNF-α using G418 after 2 weeks, as shown by a screening test; these stably transfected cells were used for the for following experiments. RT-PCR and western blot analysis indicated that autocrine TNF-α mRNA and protein levels were markedly inhibited in the siRNA-transfected SGC-996 (SGC-996si) cells. Semiquantitative analysis revealed that the TNF-α mRNA and protein expression in the SGC-996si group was markedly suppressed (Fig. 4).

### Autocrine TNF-α protein levels in the untransfected SGC-996, TNF-α siRNA-transfected SGC-996 and SGC-996NC cell culture supernatants

**Figure 5:**
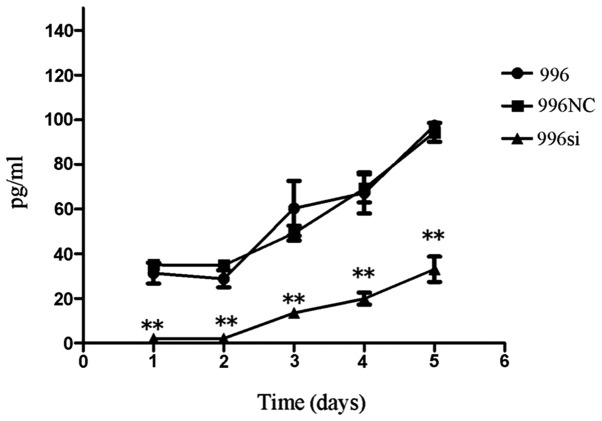
Effects of siTNF-α on autocrine tumor necrosis factor-α (TNF-α) in the SGC-996 cell line. ^*^P<0.05; ^**^P<0.05. 996si, SGC-996 cells transfected with siRNA targeting TNF-α; 996NC, SGC-996 cells transfected with negative control; 996, untransfected SGC-996 cells.

We then analyzed the autocrine TNF-α protein levels in all cell culture supernatants of the untransfected SGC-996, TNF-α siRNA-transfected SGC-996 and SGC-996NC-transfected cells (Fig. 5). Consistent with the mRNA levels, ELISA analysis revealed that when compared to the untransfected SGC-996 and SGC-996NC-transfected cells, the TNF-α siRNA-transfected SGC-996 cells showed a marked inhibition in the production of autocrine TNF-α protein. By contrast, no transfection (SGC-996) or transfection with the negative control (SGC-996NC) had no effect on autocrine TNF-α protein levels (P<0.05).

### Knockdown of TNF-α decreases the proliferation of SGC-996 cells in vitro

**Figure 6:**
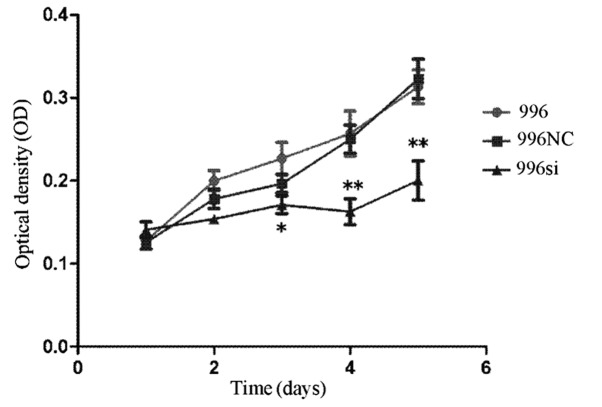
Effects of siTNF-α on the viability of untransfected SGC-996, SGC-996NC-transfected and SGC-996si cells. Proliferation ability of the SGC-996, SGC-996NC and SGC-996si cells (^*^P<0.05; ^**^P<0.05). TNF-α, tumor necrosis factor-α; 996NC, SGC-996 cells transfected with negative control; 996, untransfected SGC-996 cells.

To determine whether the endogenous TNF-α promotes cancer cell proliferation, we first treated the human SGC-996 cells with siRNA directed against TNF-α. We transfected the SGC-996 cells with TNF-α siRNA to induce the downregulation of TNF-α gene expression with C-N/siRNA and mock-treated groups were used as controls. We examined cell proliferation at 1, 2, 3, 4 and 5 days by MTT assay. Compared to the C-N/siRNA-transfected cells and the untransfected cells, cell proliferation in the TNF-α siRNA-transfected group was slower (P<0.05) (Fig. 6). The data from the *in vitro* cell proliferation assay indicated that the growth of the cells in the TNF-α siRNA-transfected group was markedly reduced compared with the untreated group, which was the same as the growth of the C-N/siRNA-transfected group. These data support the autocrine role of TNF-α in affecting the proliferaton of gallbladder cancer cells.

### TNF-α knockdown infuences gallbladder cancer cell migration and invasiveness

**Figure 7:**
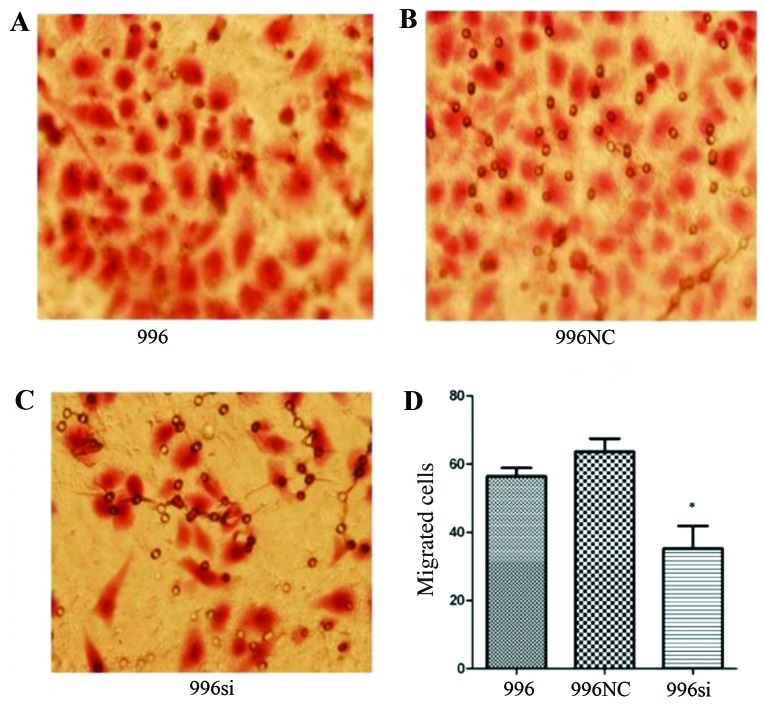
Effect of siTNF-α on SGC-996 cell migration. (A) Migration ability of untransfected SGC-996 (966) cells. (B) Migration ability of SGC-996NC-transfected (996NC) cells. (C) Migration ability of SGC-996 cells transfected with siRNA against TNF-α (996si). (D) Numbers of migrated cells in all 3 cell groups (^*^P<0.05). TNF-α, tumor necrosis factor-α.

**Figure 8:**
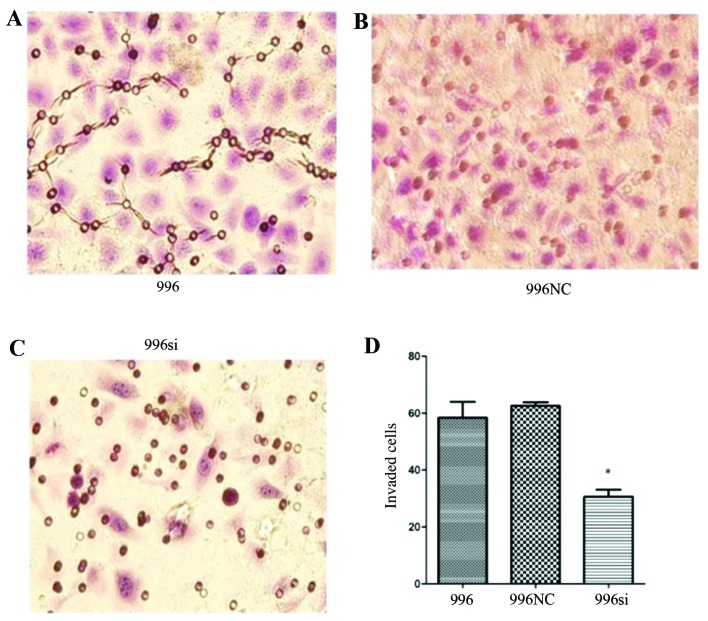
Effect of siTNF-α on SGC-996 cell invasion. (A) Invasion ability of untransfected SGC-996 (996) cells. (B) Invasion ability of SGC-996NC-transfected (996NC) cells. (C) Invasion ability of SGC-996 cells transfected with siRNA against TNF-α (996si). (D) Numbers of invaded cells in all 3 cell groups. (^*^P<0.05). TNF-α, tumor necrosis factor-α.

To determine whether the migration and invasiveness of the SGC-996 cells depends on endogenously secreted TNF-α, we used Transwell assay to examine the effects of the knockdown of the TNF-α gene in the SGC-996 cells. Following staining with H&E, 5 different fields (×400, magnification) were counted to test the numbers of migrated and invaded cells. The total number of cells in the TNF-α siRNA group that migrated and invaded through the Transwell polycarbonate filter was significantly lower than that of the cells in the SGC-996 (untreated) group (Figs. 7 and 8) (P<0.05), which was similar to the number of cells in the C-N/siRNA group. These data suggest that the function of gallbladder cancer cell-derived TNF-α plays an important role in the migration and invasion of gallbladder cancer cells.

### TNF-α knockdown does not increase the apoptosis of SGC-996 cells

**Figure 9:**
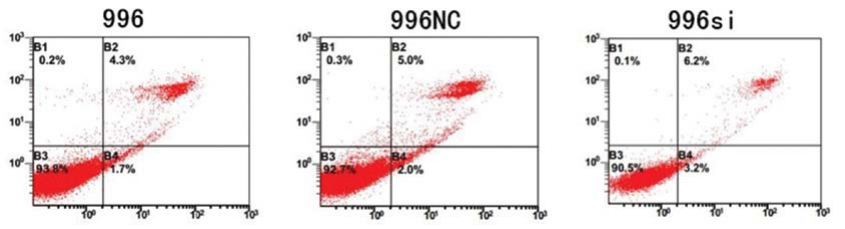
Effects of siTNF-α on the apoptosis of the untransfected SGC-996 (996), TNF-α-siRNA-transfected SGC-996 (996si) and SGC-996NC-transfected (996NC) cells as assessed by flow cytometry (FCM). The plasmid vectors were pGPU-green fluorescent protein (GFP)-siTNF-α and pGPU-GFP-NC, expressing GFP; we stained the transfected cells using red fluorescence Annexin V-PE/7-aminoactinomycin D to show the apoptotic stages, and data acquisition was achieved by FCM. SGC-996, SGC-996NC and SGC-996si TNF-α cells exhibited a similar rate of apoptosis. TNF-α, tumor necrosis factor-α.

To investigate whether the induced effects of TNF-α gene silencing on cell viability were due to apoptosis, we employed flow cytometry (FCM) after the cells were stained with Annexin V-PE/7-aminoactinomycin D. The untransfected SGC-996, TNF-α siRNA-transfected SGC-996 cells and the SGC-996NC-transfected cells exhibited a similar rate of apoptosis (Fig. 9).

### TNF-α knockdown decreases the activity of the TNF-α-AKT-NF-κB-Bcl-2 pathway, but does not promote the occurrence of apoptosis

**Figure 10:**
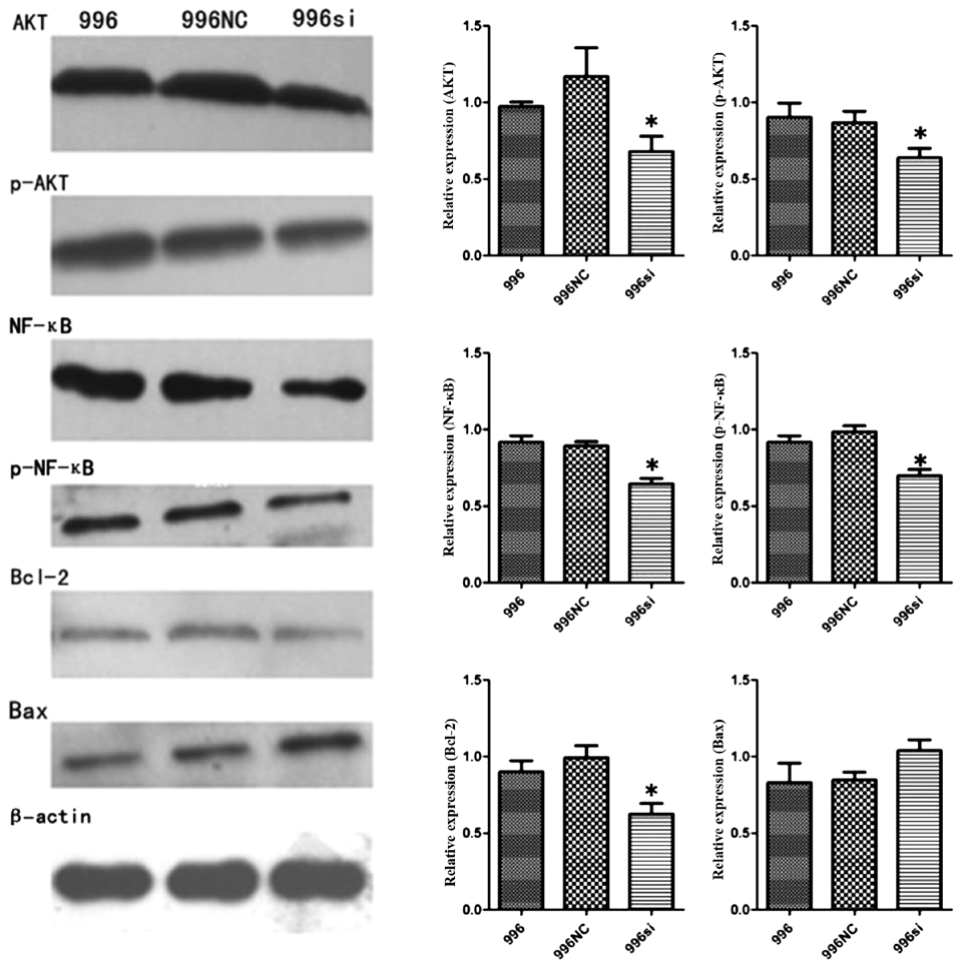
Western blot analysis indicated that the silencing of autocrine tumor necrosis factor-α (TNF-α) significantly inhibited the expression of AKT, p-AKT, nuclear factor-κB (NF-κB) (p65), p-NF-κB (p-p65) and Bcl-2 protein in the SGC-996si cells, while TNF-α silencing did not significantly increase the expression of the Bax gene. Semiquantitative analysis showed that TNF-α mRNA and protein expression in the SGC-996si group was markedly suppressed. The densitometric value for both groups was normalized to the internal control and relative expression with the following equation: normalization of both groups/the first normalized value. Data represent the means ± standard deviation (SD), n=3; ^*^P<0.05. 996si, SGC-996 cells transfected with siRNA targeting TNF-α; 996NC, SGC-996 cells transfected with negative control; 996, untransfected SGC-996 cells.

To investigate the mechanisms of TNF-α silencing responsible for the decrease in growth and invasion, we assessed the changes in AKT, p-AKT, NF-κB (p65), p-NF-κB (p-p65), Bcl-2 and Bax protein levels. These proteins are vital to the survival of gallbladder cancer cells. The western blot analysis results indicated that the AKT, p-AKT, NF-κB (p65), p-NF-κB (p-p65) and Bcl-2 protein levels in the SGC-996si cells decreased (P<0.05) in comparison to the SGC-996NC-transfected and untransfected SGC-996 cells. We also examined the expression of the Bax gene, which can promote the apoptosis of gallbladder cancer cells, and found that TNF-α silencing did not significantly increase the expression of the Bax gene P>0.05 (Fig. 10).

## Discussion

In 1975, Carswell found a factor that can rapidly cause hemorrhaging and tumor necrosis, named TNF. Thus, TNF was initially identified and named as such, as it can cause tumor necrosis (26). TNF-α, a very important inflammatory cytokine with diverse biological acitivities, such as the maintenance and homeostasis of host defence and the immune system, has been shown to be involved in malignant disease. TNF-α can promote cancer cell proliferation and invasion and it is mainly produced by macrophages. Cellular responses to TNF-α are mediated through its receptors, TNFR1 and TNFR2. The expression of each receptor is independently regulated on the surface of cells. The ability of TNF-α receptors to interact with both identical and different downstream signaling pathways explains their respective functions. TNF-α activates pathways leading to different cell functions (27), such as cell survival and proliferation, expression of inflammatory genes and cell death. TNFR1 can signal each of these biological effects and plays a crucial role in cell survival and proliferation through the pathways of NF-κB and AP-1 (28,29).

To our knowledge, this study is the first to demonstrate the role of tumor-derived TNF-α in promoting the invasion and proliferation of human gallbladder cancer cells. We further investigated the possible mechanisms underlying this process. We determined whether TNF-α was expressed in two different gallbladder cancer cell lines (NOZ and SGC-996). We found that the mRNA and protein expression levels of TNF-α in the SGC-996 cells were higher compared to the levels in the NOZ cells. Thus, on the basis of our findings and those of previous studies (5,11,23,24), tumor-derived TNF-α may play an important role in gallbladder tumor cell proliferation and invasion.

We examined cell proliferation t 1, 2, 3, 4 and 5 days by MTT assay. Compared with the C-N/siRNA and untreated groups, cell proliferation in the TNF-α siRNA group was slower. The data from the *in vitro* cell proliferation assay indicated that the growth of the cells in the TNF-α siRNA group was markedly reduced compared with the untreated group and the C-N/siRNA group. This result is different from that of a previous study on ovarian cancer, in which the authors concluded that TNF-α RNAi cells grew at a similar rate to normal cells (11). We repeated our experiment and found that the results were consistent in the gallbladder cancer cells. The reasons for this phenomenon require further investigation.

A number of studies have reported that TNF-α can induce the expression of MMPs, interleukin (IL)-8, CXC chemokine receptor type 4 (CXCR), VEGF and MCP-1, thus enhancing tumor cell invasion and metastasis (17,18,20). Our research team also found that CXCR and VEGF-C/D promote gallbladder cancer cell proliferation and invasion (30–32). In this study, we found that the migration and invasion ability was inhibited when the TNF-α gene was silenced *in vitro*. This suggests that tumor-derived TNF-α exerts a profound effect on migration and invasion. As we used the RNAi technology to silence the TNF-α gene in SGC-996 cells, the expressoin of the cytokines, MMPs, IL-8, CXCR, VEGF and MCP-1, was decreased in the gallbladder cells (data not shown). In the present study, to determine whether the suppressive effects of TNF-α gene silencing on cell viability were due to apoptosis, we employed flow cytometry after the cells were stained with red fluorescence Annexin V-PE/7-aminoactinomycin D. The untransfected SGC-996, TNF-α siRNA-transfected SGC-996 and SGC-996NC-transfected cells exhibited a similar rate of apoptosis. Further studies are required to elucidate the specific mechanisms responsible for this phenomenon.

In this study, we assessed the changes in AKT, p-AKT, NF-κB (p65), p-NF-κB (p-p65), Bcl-2 and Bax protein expression. These proteins are vital to the survival of gallbladder cancer cells. The western blot analysis results indicated that AKT, p-AKT, NF-κB (p65), p-NF-κB (p-p65) and Bcl-2 protein levels in the SGC-996si cells were decreased compared with those in the SGC-996NC-transfected and untransfected SGC-996 cells. We also examined the expression of Bax, which can promote the apoptosis of gallbladder cancer cells, and found that TNF-α silencing did not significantly increase the expression of the Bax gene.

In conclusion, in the present study, we verify the biological behavior of gallbladder cancer cell-derived TNF-α. We provide evidence that the reduction of autocrine TNF-α in gallbladder cancer cells can exert inhibitory effects on the ability of the cells to grow and migrate *in vitro*. This provides further evidence that targeting TNF-α and its intracellular pathways may prove useful in the treatment of gallbladder cancer.
